# Chinese expert consensus on multidisciplinary diagnosis and treatment of hepatocellular carcinoma with portal vein tumor thrombus: 2016 edition

**DOI:** 10.18632/oncotarget.12817

**Published:** 2016-10-21

**Authors:** Cheng Shuqun, Chen Minshan, Cai Jianqiang, The National Research Cooperative Group for Diagnosis

**Affiliations:** ^1^ Eastern Hepatobiliary Surgical Hospital, Second Military Medical University, Shanghai, China; ^2^ Department of Hepatobiliary Surgery, Sun Yat-sen University Cancer Center, Guangzhou, China; ^3^ Department of Hepatobiliary Surgery, Cancer Hospital, Chinese Academy of Medical Sciences, Beijing, China

**Keywords:** hepatocellular carcinoma, portal vein tumor thrombosis, consensus, China

## Abstract

Hepatocellular carcinoma is the fourth leading cause of cancer-related morbidity and mortality in China. Portal vein tumor thrombus (PVTT) is common and it worsens prognosis of hepatocellular carcinoma (HCC). There is no internationally accepted consensus or guideline for diagnosis and treatment of HCC with PVTT. Based on existing evidences and common current practices, Chinese Experts on Multidisciplinary Diagnosis and Treatment of HCC with portal vein tumor thrombus met to develop a national consensus on diagnosis and treatment of HCC with PVTT. The meeting concluded with the First Edition (version 2016) of consensus statements with grades of evidence given as grades Ia, Ib, IIa, IIb, III and IV, and ranking as Classes A, B, C, D and I for quality of evidence and strength of recommendation by the United State Preventive Service Task Force, respectively. The consensus suggests recommended treatment to be based on patients PVTT type and ECOG functional status; surgery being the preferred treatment for Child-Pugh A, PVTT type I/II, and ECOG PS 0-1; transcatheter arterial chemoembolization (TACE) for non-resectable PVTT I/II and Child-Pugh A; and radiotherapy for non-resectable PVTT I/II/III and Child-Pugh A. Symptomatic treatment is recommended for Child-Pugh C, with massive ascites or gastrointestinal bleeding. By updating clinicians with treatment options for HCC with PVTT, the consensus statement aimed to prolong overall survival and to improve quality of life of patients with minimal treatment complication. Future treatment strategies for HCC with PVTT in China would depend on new evidences from more future clinical trials, especially studies defining the role of traditional Chinese medicine and clarifying molecular aspects of HCC.

## INTRODUCTION

Hepatocellular carcinoma (HCC) is the sixth most prevalent cancer worldwide, and China accounts for more than half of new cases and deaths related to HCC every year [1]. The latest data indicated that the morbidity and mortality rates of HCC ranked the fourth and third, respectively, among all malignant tumors reported in China [2]. Given the advances in diagnosis and treatment strategies for different stages of HCC, the prognosis of HCC patients has improved. Unfortunately, 70% to 80% of patients are still diagnosed at an advanced stage as there are no obvious clinical symptoms at early stages. At present, the overall prognosis of HCC is not satisfactory.

Owing to the biological characteristics of liver cancer and the anatomical characteristics of the liver, HCC is prone to invade intrahepatic vessels, especially the portal venous system. In China, the incidences of portal vein tumor thrombus (PVTT) have been reported to range from 44% to 62.2% [3]. Once developed, PVTT progresses rapidly to cause portal hypertension, hepatocellular jaundice, and intractable ascites. The median survival of HCC patients with main PVTT is 2.7 months [4]. PVTT plays a major role in the prognosis and clinical staging of HCC [5, 6].

There have been no worldwide consensuses or guidelines on the diagnosis and treatment of HCC with PVTT. Guidelines in Europe and America follow the Barcelona Clinic Liver Cancer Staging (BCLC) and regard HCC with PVTT to be at BCLC Stage C. The guidelines also recommend treating HCC patients with PVTT with sorafenib alone [7]. On the contrary, experts from Southeast Asian countries opine that multidisciplinary therapy including surgery, transcatheter arterial chemoembolization (TACE), radiotherapy, and/or sorafenib should be considered to achieve more satisfactory outcomes [8]. Consequently, the Chinese National Research Cooperative Group for Diagnosis and Treatment of Hepatocellular Carcinoma with Tumor Thrombus was set up to arrive at a national consensus on the diagnosis and treatment of HCC with PVTT, based on the existing evidences published internationally and in China. The Chinese Expert Consensus on Multidisciplinary Diagnosis and Treatment of Hepatocellular Carcinoma with Portal Vein Tumor Thrombus was finally developed after repeated meetings and modifications of the draft by top Chinese experts on HCC with PVTT. This version (version 2016) is the first edition of consensus and it will be updated regularly as new evidences become available.

Based on internationally accepted practice, the grades of evidence we use are presented in Table 1 [9]. We also adopted the United States Preventive Service Task Force (USPSTF) recommendations to assign 5 alphabets (A, B, C, D, and I) to denote the strength of recommendation for clinical practice (Table 2) [10].

**Table 1:** Grades of Evidences

Grades of Evidences	Description
Ia	Evidences are originated from the meta-analysis results of various RCTs
Ib	Evidences are originated from the results of at least one well-designed RCT
IIa	Evidences are originated from the results of at least one well-designed perspective non-RCT
IIb	Evidences are originated from the results of at least one well-designed interventional clinical research of other type
III	Evidences are originated from the well-designed non-interventional clinical researches, such as descriptive researches and relevant researches
IV	Evidences are originated from the reports made by committee of experts or the clinical reports of authoritative experts

Abbreviations: RCT, randomized controlled trial

**Table 2:** Ranking of Recommended Opinion

Grades of Evidences	Description
A	Favorable scientific evidences indicate that the medical treatment can provide clear and definite benefits to the patients; physicians are strongly recommended to administer the medical treatment to eligible patients.
B	Existing evidences indicate that the medical treatment may provide moderate benefits that outweigh the potential risks; physicians may suggest or patients may carry out the said medical treatment.
C	Existing evidences indicate that the medical treatment may provide only little benefits, or the benefits do not outweigh the risks; physicians may suggest or administer the said medical treatment selectively based on the patient's condition.
D	Existing evidences indicate that the medical treatment would not benefit the patients, or the potential risks would outweigh the benefits; physicians are recommended not to administer the said medical treatment in patients.
I	There are not enough scientific evidences, or the existing evidences cannot be used, to evaluate the benefits and risks of the said medical treatment; physicians should help the patients understand well the uncertainty of this medical treatment.

## CONSENSUS RECOMMENDATIONS

### Diagnosis and Classification of PVTT

PVTT is one of the most common complications of HCC [11]. A diagnosis of HCC is a prerequisite to diagnose PVTT [12]. The imaging features of PVTT include solid lesions within the portal vein in all the phases of intravenous enhanced three-phase computed tomography (CT), especially with enhancement of contrast in the arterial phase and washout in the portal venous phase of the procedure [13, 14]. Clinically, PVTT should be distinguished from portal vein thrombosis (PVT), which occurs as a complication of cirrhosis or after splenectomy. PVT is not enhanced in the arterial phase. It occasionally disappears or improves after anticoagulant therapy [15].

The extent of PVTT is closely related to prognosis of HCC. The HCC staging systems that are commonly used today are the TNM staging, BCLC staging, and Japanese integrated staging (JIS) systems. All these staging systems accept the importance of PVTT. However, they do not further define the extent of PVTT. At present, there are two classifications for PVTT: the Japanese V_P_ classification [16], and the Cheng's classification as suggested by Professor Cheng Shuqun of China [17–19].

The Cheng's classification comprises four levels based on the extent of tumor thrombus in the portal vein shown on medical imagings: type I, tumor thrombus involving segmental or sectoral branches of the portal vein or above; type II, tumor thrombus involving the right/left portal vein; type III, tumor thrombus involving the main portal vein; and type IV, tumor thrombus involving the superior mesenteric vein. Type I_0_, tumor thrombus found only under microscopy. Many studies have supported that the Cheng's classification to be more applicable than the V_P_ classification for disease assessment, treatment selection, and prognostic judgment in patients with PVTT [18–20], and hence it is recommended to be used for classifying the extent of PVTT.

## MULTIDISCIPLINARY THERAPY (MDT) PATH FOR HCC WITH PVTT

**Figure 1:**
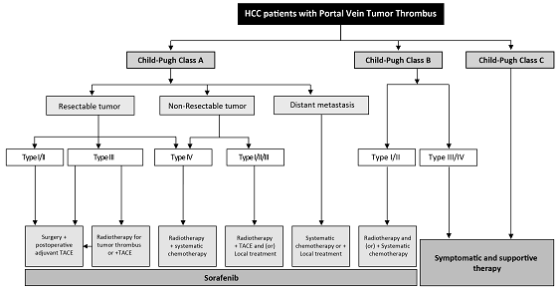
Diagnosis and treatment of HCC with PVTT

A multidisciplinary team to coordinate diagnosis and treatment of HCC patients with PVTT provides maximal benefits to patients. The therapeutic plan for the treatment of HCC with PVTT formulated by the National Research Cooperative Group for Diagnosis and Treatment of Hepatocellular Carcinoma with Tumor Thrombus is presented in Figure 1. Patients with Child-Pugh A liver function can undergo any treatment according to the PVTT type. When the lesion is resectable and when there is no extrahepatic metastasis, patients with type I/II PVTT should undergo surgical resection of the PVTT en bloc with the primary HCC. For patients with PVTT type III, the treatment choices include surgery, radiotherapy, and/or TACE depending on the patient's preference. For unresectable lesions, patients with type I/II PVTT should receive radiotherapy combined with TACE as the primary treatment, and patients with type III and IV PVTT should receive radiotherapy or systemic therapy. Patients with Child-Pugh B liver function should first receive antiviral treatment for HCC secondary to hepatitis B or C infections. If the liver function improves to Child-Pugh A, then these patient subgroups can be treated as mentioned above. Surgery and TACE are not recommended for Child-Pugh B patients. Child-Pugh C patients should only receive supportive care. Child-Pugh A and Child-Pugh B patients who have extrahepatic metastases can receive systemic chemotherapy and/or local treatment. Sorafenib can be used for patients with all extents of PVTT with Child-Pugh A and B liver function.

### Recommended first-line treatment options for PVTT

The treatment of HCC patients with PVTT is based on the patients’ liver function, the stage of hepatic lesion, and the extent of PVTT. A strategy that can either eliminate or control HCC with PVTT using multimodality therapy can extend survival and improve quality of life of the patient.

## SURGERY

### Recommendations

- Surgery is the preferred treatment in patients with Child-Pugh A, PVTT type I/II, and ECOG PS 0-1 (Evidence level IIb, Recommendation A); type III PVTT patients can undergo surgery directly or after tumor downstaging using radiotherapy and/or TACE (Evidence level IIb, Recommendation B).

Surgical treatment is considered to be potentially curative and is the preferred treatment option for HCC patients with type I/II PVTT. En bloc resection of the primary HCC and PVTT provides a potential for cure. Many studies reported that patients who had undergone surgery had better prognosis than those treated with TACE [12, 21, 22].

Type I/II PVTT are more suitable for resection than type III/IV (Evidence level IIb) [18, 23]. En-bloc resection can be performed in type I/II PVTT patients with partial hepatectomy or hemi-hepatectomy[11]. For type III PVTT patients, as the PVTT has extended to the main portal vein, partial hepatectomy has to be combined with thrombectomy or main portal vein resection followed by reconstruction. At present, studies have revealed that there is no significant difference in prognosis among these surgical procedures (Evidence level IIb) [24]. Thrombectomy is by far the most commonly used surgical procedure. In the study based on the findings of the Japanese registry, patients with type III/IV PVTT (extended to the main portal vein or the contralateral branch) and beyond had no significant improvement in survival after surgical treatment. On the other hand, patients with I/II PVTT had significantly improved mean survival when compared to patients not undergoing surgical treatment (2.87 years vs. 1.10 years; diff: 1.77 years, *P* < 0.001) [25]

The following are the recommendations for reducing recurrence rates and metastasis after surgery: (1) Pre-operative small-dose radiotherapy has been reported to downstage some type III PVTT patients, reduce recurrence rate without increasing surgical risks, and reduce postoperative hepatic failure rates (Evidence level IIa) [26]. (2) Although adjuvant TACE after surgery has been reported to reduce recurrence rates and prolong survival of PVTT patients in a randomized controlled trial [27] and in a retrospective study [28], the Japanese nationwide survey failed to support the use of adjuvant TACE [25].

Other treatment recommendations that are controversial include the following: (1) Pre-operative TACE has been reported to improve postoperative survival, but it may increase operative risks (Evidence level IIb) [29]. (2) There is a lack of high-level evidence for adjuvant radiotherapy or chemotherapy.

## NONSURGICAL THERAPIES

### Transhepatic Arterial Infusion (TAI) or TACE

#### Recommendations

- Patients with non-resectable primary tumor, type I/II PVTT, and Child-Pugh A liver function may receive TACE (Evidence level IIb, Recommendation B) alone or in combination with radiotherapy (Evidence level IIb, Recommendation A).
- Patients with Child-Pugh B liver function or type III/IV PVTT are not recommended to receive TACE (Evidence level IIb, Recommendation C).

TACE/TAI is one of the most commonly used techniques to manage nonresectable HCC with PVTT [22]. Despite the possible benefit of TACE in prolonging overall survival (4-7 months) in patients with HCC and PVTT type III/IV, the use of TACE in patients is controversial due to the risk of liver infarction and hepatic failure [30]. At present, TACE is considered for PVTT patients with good liver function with adequate collateral circulation around the obstructed portal vein [31, 32]. The overall survival rate varies greatly among patients with PVTT after TACE. The patient survival rates decreased from 82% at 3-months to 71% at 6 months and 47% at 12 months, with a median survival of 10 months. Patients with Child-Pugh A liver function had better median survival when compared to patients with Child-Pugh B (15 months vs. 13 months) [33], and the complete remission rate (CR), partial remission rate (PR), and stable disease rate (SD) were reported to be 0, 19.5% to 26.3%, and 42.5% to 62.7%, respectively[34, 35, 36]. Lipiodol and gelatin sponge are common embolizing agents used in TACE [37]. Some reports have suggested that TACE, when combined with lipiodol, is more effective than TAI or conservative treatment [22, 38]. The effectiveness of the embolizing agents depends on their size. The smaller the diameter of an embolizing agent, the better is the effect on PVTT patients and the lower is its adverse side effects [39, 40]. The use of super-selective catheterization improves therapeutic effects and reduces damages to the normal liver when compared with conventional TACE. Recently, TACE with drug-eluting beads has been introduced into clinical application; however, its effects on HCC patients with PVTT are controversial [41].

### Radiotherapy (RT)

### External beam radiation therapy

#### Recommendations

- Patients with nonresectable HCC with all types of PVTT, with Child-Pugh A or B liver function, are recommended to receive RT with the target region containing both the primary tumors and PVTT - 3DCRT or IMRT 95% PTV 40-60 Gy/2-3 Gy (Evidence level IIb, Recommendation B) or SBRT 36-40 Gy/5-6 Gy (Evidence level IIb, Recommendation A).
- Patients with Child-Pugh A liver function and type I, II, and III PVTT are recommended to receive combined radiotherapy and TACE (Evidence level IIb, Recommendation A). The radiotherapy target region includes the primary tumor and PVTT or only the PVTT.

With development of newer technologies such as three-dimensional conformal radiotherapy (3DCRT), intensify-modulated radiotherapy (IMRT), and three-dimensional oriented radiotherapy (SBRT), radiation dosage to the targeted regions can be increased while giving better protection to the adjacent healthy tissues [42–44]. This allows the maximum use of radiotherapy technologies and enables their use in HCC patients with all types of PVTT. The use of radiotherapy alone or in combination with other treatment such as TACE improved survival and quality of life in HCC patients with PVTT [44].

Target localization suggests the use of CT and MRI image fusion technology based on the area of lipiodol deposition after TACE. The clinical target volume (CTV) is 5 to 10 mm larger than the diameter of the tumor area. The plan target volume (PTV) should be determined on the basis of a moving target, set-up error, and random error. The designation of the irradiation area is still controversial, which should be determined individually. The hepatic lesion and PVTT should be irradiated simultaneously if the hepatic lesion is small and PVTT is nearby. If the volume of the primary tumor is large or PVTT is distant to the primary tumor, only the PVTT should receive irradiation [45].

There is not enough evidence to determine the best radiation and fraction doses. The existing evidence suggests a positive correlation between total radiation dose and tumor response [46]. However, multivariate analysis only showed response to radiotherapy to be associated with survival [46, 47].

Radiation-induced liver disease (RILD) or radiation hepatitis is a subacute form of liver injury, which occurs due to over exposure of the liver to radiation [48]. The key to prevent RILD is to keep the total dose within the tolerance range limit when designing the radiotherapy plan [48]. As most HCC patients in China have a cirrhotic background, the radiation tolerance dose of the liver in these patients is lower than that in patients from other countries. The liver tolerance dose (average dose of the liver) is 23 Gy for Child-Pugh A patients and only 6 Gy for Child-Pugh B patients [49]. The most common risk factors of RILD include pre-existing poor liver function, high irradiation volume, coexisting PVT and acute liver toxicity due to other causes [48, 49]. Evidence from clinical studies has shown a combination of radiotherapy and TACE produces better clinical outcomes than TACE or radiotherapy alone. The time interval between TACE and radiotherapy should not exceed 1 month [50]. When TACE is combined with radiotherapy, the order of the treatments given should be decided clinically. As the effect on liver function is less in patients receiving radiotherapy first than those receiving TACE first, with similar treatment outcomes, radiotherapy should be given before TACE [44].

### Internal Radiation Therapy

#### Recommendations

- Patients with nonresectable primary tumors; type I, II, and III PVTT; and Child-Pugh A liver function should be treated with TARE (Evidence level IIb, Recommendation B) or portal veins I^125^ seed implantation (Evidence level IIb, Recommendation B).

Patients treated with I^125^ particle seeds implanted in the portal vein and TACE have been reported to have better survival outcomes when compared to patients treated with TACE alone. This combination therapy also improved the reperfusion rate of portal vein significantly [51]. Another study showed I^125^ seeds followed by TACE significantly improved the median survival and progression free survival rates when compared to I^125^ alone (*P* = 0.037 and 0.002, respectively) [52]. Transarterial arterial radio-embolization (TARE) with yttrium-90 (Y90) microspheres is considered to be a viable treatment option in HCC patients with PVTT. TARE has been shown to produce better long-term survival outcomes than TACE [53]. Furthermore, patients treated with TARE required shorter periods of hospitalization when compared to TACE [54]. However, there is no uniform dosage standard at present for internal radiation therapy.

### Systematic Therapy

#### Recommendations

- Nucleoside analogs are recommended in patients with PVTT with positive HBV-DNA (Evidence level 1a, Recommendation A). Re-activation of HBV is of high importance in patients detected with negative HBV-DNA.
- Sorafenib is recommended as the basic drug for PVTT patients (Evidence level Ib, Recommendation A).
- Chemotherapy is recommended in PVTT patients (Evidence level IIb, Recommendation B) with extrahepatic metastasis and Child-Pugh A or B liver function.

Persistent HBV infection is an important poor risk factor for occurrence, progression, recurrence, and death in patients with HCC secondary to HBV infection. Antiviral therapy reduces postoperative recurrence and improves survival of HCC patients [55]. Antiviral therapy should also be given to PVTT patients[56, 57].

Sorafenib is a universally accepted therapy that effectively prolongs survival in patients with advanced HCC (Evidence level Ib) [58]. Sorafenib has been listed by the China Food and Drug Administration (CFDA) as the first-line treatment option in patients with advanced HCC. The STORM, was a phase 3, double-blind, randomized, placebo-controlled study, which evaluated the effectiveness of sorafenib as adjuvant therapy to surgery. When compared to placebo, sorafenib did not show any significant improvement in the median recurrence-free survival (33.3 months vs. 33.7 months, *P* = 0.26), suggesting that adjuvant sorafenib to be ineffective [59]. The effectiveness of Sorafenib and TACE combination has also been controversial [25, 60, 61].

The EACH study demonstrated that FOLFOX 4 (an oxaliplatin-containing chemotherapy) provided partial cure in patients with advanced HCC (including PVTT patients). FOLFOX 4 might be administered in patients with good liver function and tolerance (Evidence level Ib) [62].

### Local Treatment

#### Recommendations

- Local ablation therapies should be recommended in PVTT patients with caution; further studies are warranted (Evidence level III, Recommendation C). Local ablation therapies may be combined with TACE (Evidence level IIb, Recommendation B).

Local treatment of PVTT includes local ablation therapies and portal venous stenting. The local ablation therapies include percutaneous ethanol injection (PEIT), radiofrequency ablation (PRFA), and laser ablation (LA). These therapies may be adopted to reduce tumor load and recanalization of portal vein. However, local therapies must be used cautiously as there is a risk of damaging the portal vein wall and bile duct. In addition, a high recurrence rate of PVTT has been reported within a short period of time (Level III evidence) [63, 64]. Portal vein stenting may be adopted to recanalize blood flow in the portal veins of PVTT patients, with resultant increase in blood flow to the liver, but without reducing the tumor load. In patients with PVTT, portal vein stenting can result in improved liver functions, reduced portal vein pressure, and at the same time, win time for other therapies such as radiotherapy and TACE to act (Evidence level III) [65].

### Symptomatic and Supportive Treatment

#### Recommendations

- Symptomatic and supportive treatment is recommended in patients with Child-Pugh C liver function, with massive ascites or gastrointestinal bleeding due to esophageal varices and hepatic encephalopathy (Evidence level Ia, Recommendation A).

Most complications of PVTT result from portal hypertension. The common complications include upper gastrointestinal hemorrhage, ascites, hypersplenism, hepatorenal syndrome, and hepatic failure. For therapeutic methods, please refer to the article on treatment of portal hypertension [66].

## FUTURE OUTLOOK

It is necessary to develop a treatment consensus in China as HCC patients with PVTT in China are different from those in Europe and America in terms of etiology and biological behavior. Although treatment of HCC patients with PVTT is still controversial, new evidences are being gathered. Similar to the multidisciplinary approach of HCC treatment in the United States (the American Association for the Study of Liver Diseases practice guidelines) and Europe (the European Association for the Study of the Liver - European Organization for Research and Treatment of Cancer) for HCC management, we have adopted a multidisciplinary approach for HCC with PVTT. This treatment approach when combined with early diagnosis, will enable a larger number of patients to receive an appropriate treatment based on the stage of the disease.

In our consensus meetings, the following principles in clinical practice are emphasized: (1) Multidisciplinary treatment should be used in HCC patients with PVTT to achieve better results. (2) Prolongation of overall survival is the most important target and the chance of cure is low. Emphasis should also be given to the quality of life of these patients. The treatment complication rate should be kept at a minimum. (3) Local treatment should be combined with systemic treatment to provide better long-term survival for these patients.

More RCTs should be conducted in HCC patients with PVTT. The molecular mechanisms underlying the genesis and development of PVTT also need to be studied to lay the foundation of more future effective treatment. The role of Chinese traditional medicine in the treatment of PVTT as an adjuvant to other therapeutic options such as surgical treatment, TACE, or radiotherapy should be evaluated.

Members of the National Research Cooperative Group for Diagnosis and Treatment of Hepatocellular Carcinoma with Tumor Thrombus*

Cheng Shuqun; Chen Minshan; Cai Jianqiang; Wu Mengchao; Tang Zhaoyou; Lau WanYee; Wang Xuehao; Zheng Shusen; Chen Xiaoping; Wang Hongyang; Bi Xinyu; Bie Ping; Cai Xiujun; Cao Jianping; Chen Guihua; Chen Jisheng; Chen Yajin; Cheng Hongyan; Cong Wenming; Dai Chaoliu; Dong Jiahong; Dou Kefeng; Fan Jia; Fang Chihua; Geng Xiaoping; Guo Rongping; Han Guohong; Hong Defei; Huo Feng; Jia Weidong; Jiang Hongchi; Jin Jing; Li Gong; Li Lequn; Li Bin; Li Bo; Li Huai; Li Jun; Li Qiang; Li Zhiyu; Liang Lijian; Liu Jingfeng; Liu Lianxin; Liu Yingbin; Lu Shichun; Ma Kuansheng; Mao Yilei; Meng Qinghua; Meng Yan; Meng Zhiqiang; Peng Baogang; Peng Shuyou; Peng Zhihai; Qin Lunxiu; Qiu Yudong; Ren Zhenggang; Shen Feng; Sun Juxian; Teng Gaojun; Wang Lu; Wang Yi; Wen Tianfu; Wu Liqun; Xia Feng; Xia Jinglin; Xing Baocai; Xu Li; Xu Xiao; Yang Dinghua; Yang Guangshun; Yang Jiamei; Yang Lianyue; Yang Yang; Yang Yefa; Ye Shenglong; Ying Mingang; Zeng Zhaochong; Zhang Bixiang; Zhang Qi; Zhao Hong; Zheng Yaxin; Zhou Aiping; Zhou Jian; Zhou Jie; Zhou Weiping; Zhou Xinda.

## References

[1] Torre LA, Bray F, Siegel RL, Ferlay J, Lortet-Tieulent J, Jemal A Global cancer statistics, 2012. CA Cancer J Clin.

[2] Chen W, Zheng R, Baade PD, Zhang S, Zeng H, Bray F, Jemal A, Yu XQ, He J Cancer statistics in China, 2015. CA Cancer J Clin.

[3] Zhang ZM, Lai EC, Zhang C, Yu HW, Liu Z, Wan BJ, Liu LM, Tian ZH, Deng H, Sun QH, Chen XP The strategies for treating primary hepatocellular carcinoma with portal vein tumor thrombus. Int J Surg.

[4] Pawarode A, Voravud N, Sriuranpong V, Kullavanijaya P, Patt YZ Natural history of untreated primary hepatocellular carcinoma: a retrospective study of 157 patients. Am J Clin Oncol.

[5] Li SH, Wei W, Guo RP, Shi M, Guo ZX, Chen ZY, Xiao CZ, Cai MY, Zheng L Long-term outcomes after curative resection for patients with macroscopically solitary hepatocellular carcinoma without macrovascular invasion and an analysis of prognostic factors. Med Oncol.

[6] Li SH, Guo ZX, Xiao CZ, Wei W, Shi M, Chen ZY, Cai MY, Zheng L, Guo RP Risk factors for early and late intrahepatic recurrence in patients with single hepatocellular carcinoma without macrovascular invasion after curative resection. Asian Pac J Cancer Prev.

[7] Bruix J, M; Sherman (2011). American Association for the Study of Liver Diseases. Management of hepatocellular carcinoma: an update Hepatology.

[8] Cheng S, Yang J, Shen F, Zhou W, Wang Y, Cong W, Yang GS, Cheng H, Hu H, Gao C, Guo J, Li A, Meng Y (2016). Multidisciplinary management of hepatocellular carcinoma with portal vein tumor thrombus - Eastern Hepatobiliary Surgical Hospital consensus statement. Oncotarget.

[9] Ryder SD (2003). British Society of Gastroenterology. Guidelines for the diagnosis and treatment of hepatocellular carcinoma (HCC) in adults. Gut.

[10] U.S. Preventive Services Task Force (2016). Grade Definitions and Suggestions for Practice. http://www.uspreventiveservicestaskforce.org/Page/Name/grade-definitions.

[11] Shaohua L, Qiaoxuan W, Peng S, Qing L, Zhongyuan Y, Ming S, Wei W, Rongping G Surgical Strategy for Hepatocellular Carcinoma Patients with Portal/Hepatic Vein Tumor Thrombosis. PLoS One.

[12] Wang K, Guo WX, Chen MS, Mao YL, Sun BC, Shi J, Zhang YJ, Meng Y, Yang YF, Cong WM, Wu MC, Lau WY, Cheng SQ Multimodality treatment for hepatocellular carcinoma with portal vein tumor thrombus: a large-scale, multicenter, propensity mathching score analysis. Medicine (Baltimore).

[13] S; Qin Primary Liver Cancer Diagnosis and Treatment Expert Panel of the Chinese Ministry of Health. Guidelines on the diagnosis and treatment of primary liver cancer (2011 edition). Chin Clin Oncol.

[14] Hennedige T, Venkatesh SK Advances in computed tomography and magnetic resonance imaging of hepatocellular carcinoma. World J Gastroenterol.

[15] Ponziani FR, Zocco MA, Campanale C, Rinninella E, Tortora A, Di Maurizio L, Bombardieri G, De Cristofaro R, De Gaetano AM, Landolfi R, Gasbarrini A Portal vein thrombosis: Insight into physiopathology, diagnosis, and treatment. World J Gastroenterol.

[16] Ikai I, Yamamoto Y, Yamamoto N, Terajima H, Hatano E, Shimahara Y, Yamaoka Y Results of hepatic resection for hepatocellular carcinoma invading major portal and/or hepatic veins. Surg Oncol Clin N Am.

[17] Shuqun C, Mengchao W, Han C, Feng S, Jiahe Y, Guanghui D, Wenming C, Peijun W, Yuxiang Z (2007). Tumor thrombus types influence the prognosis of hepatocellular carcinoma with the tumor thrombi in the portal vein. Hepatogastroenterology.

[18] Shi J, Lai EC, Li N, Guo WX, Xue J, Lau WY, Wu MC, Cheng SQ Surgical treatment of hepatocellular carcinoma with portal vein tumor thrombus. Ann Surg Oncol.

[19] Shi J, Lai EC, Li N, Guo WX, Xue J, Lau WY, Wu MC, Cheng SQ A new classification for hepatocellular carcinoma with portal vein tumor thrombus. J Hepatobiliary Pancreat Sci.

[20] Niu ZJ, Ma YL, Kang P, Ou SQ, Meng ZB, Li ZK, Qi F, Zhao C Transarterial chemoembolization compared with conservative treatment for advanced hepatocellularcarcinoma with portal vein tumor thrombus: using a new classification. Med Oncol.

[21] Peng ZW, Guo RP, Zhang YJ, Lin XJ, Chen MS, Lau WY Hepatic resection versus transcatheter arterial chemoembolization for the treatment of hepatocellular carcinoma with portal vein tumor thrombus. Cancer.

[22] Xue TC, Xie XY, Zhang L, Yin X, Zhang BH, Ren ZG Transarterial chemoembolization for hepatocellular carcinoma with portal vein tumor thrombus: a meta-analysis. BMC Gastroenterol.

[23] Chen XP, Qiu FZ, Wu ZD, Zhang ZW, Huang ZY, Chen YF, Zhang BX, He SQ, Zhang WG Effects of location and extension of portal vein tumor thrombus on long-term outcomes of surgical treatment for hepatocellular carcinoma. Ann Surg Oncol.

[24] Chok KS, Cheung TT, Chan SC, Poon RT, Fan ST, Lo CM Surgical outcomes in hepatocellular carcinoma patients with portal vein tumor thrombosis. World J Surg.

[25] Kokudo T, Hasegawa K, Matsuyama Y, Takayama T, Izumi N, Kadoya M, Kudo M, Ku Y, Sakamoto M, Nakashima O, Kaneko S, N; Kokudo (2016). Liver Cancer Study Group of Japan. Survival benefit of liver resection for hepatocellular carcinoma associated with portal vein invasion. J Hepatol.

[26] Li N, Feng S, Xue J, Wei XB, Shi J, Guo WX, Lau WY, Wu MC, Cheng SQ, Meng Y Hepatocellular carcinoma with main portal vein tumor thrombus: a comparative study comparing hepatectomy with or without neoadjuvant radiotherapy. HPB (Oxford).

[27] Peng BG, He Q, Li JP, Zhou F Adjuvant transcatheter arterial chemoembolization improves efficacy of hepatectomy for patients with hepatocellular carcinoma and portal vein tumor thrombus. Am J Surg.

[28] Bai T, Chen J, Xie ZB, Wu FX, Wang SD, Liu JJ, Li LQ (2016). The efficacy and safety of postoperative adjuvant transarterial embolization and radiotherapy in hepatocellular carcinoma patients with portal vein tumor thrombus.Onco Targets Ther.

[29] Yoshidome H, Takeuchi D, Kimura F, Shimizu H, Ohtsuka M, Kato A, Furukawa K, Yoshitomi H, Miyazaki M Treatment strategy for hepatocellular carcinoma with major portal vein or inferior vena cava invasion: a single institution experience. J Am Coll Surg.

[30] Chan SL, Chong CC, Chan AW, Poon DM, Chok KS Management of hepatocellular carcinoma with portal vein tumor thrombosis: Review and update at 2016. World J Gastroenterol.

[31] Chung GE, Lee JH, Kim HY, Hwang SY, Kim JS, Chung JW, Yoon JH, Lee HS, Kim YJ Transarterial chemoembolization canbe safely performed in patients with hepatocellular carcinoma invading the main portal vein and may improve the overall survival. Radiology.

[32] Kim HC, Chung JW, Lee W, Jae HJ, Park JH (2005). Recognizing extrahepatic collateral vessels that supply hepatocellular carcinoma to avoid complications of transcatheter arterial chemoembolization. Radiographics.

[33] Ajit Y, Sudarsan H, Saumya G, Abhishek A, Navneet R, Piyush R, Anil A, Arun G Transarterial chemoembolization in unresectable hepatocellular carcinoma with portal vein thrombosis: a perspective on survival. Oman Med J.

[34] Liu L, Zhang C, Zhao Y, Qi X, Chen H, Bai W, He C, Guo W, Yin Z, Fan D, Han G Transarterial chemoembolization for the treatment of advanced hepatocellular carcinoma with portal vein tumor thrombosis: prognostic factors in a single-center study of 188 patients. Biomed Res Int.

[35] Jang JW, Bae SH, Choi JY, Oh HJ, Kim MS, Lee SY, Kim CW, Chang UI, Nam SW, Cha SB, Lee YJ, Chun HJ, Choi BG A combination therapy with transarterial chemo-lipiodolization and systemic chemo-infusion for large extensive hepatocellular carcinoma invading portal vein in comparison with conservative management. Cancer Chemother Pharmacol.

[36] Luo J, Guo RP, Lai EC, Zhang YJ, Lau WY, Chen MS, Shi M Transarterial chemoembolization for unresectable hepatocellular carcinoma with portal vein tumor thrombosis: a prospective comparative study. Ann Surg Oncol.

[37] Liu YS, Ou MC, Tsai YS, Lin XZ, Wang CK, Tsai HM, Chuang MT Transarterial Chemoembolization Using Gelatin Sponges or Microspheres Plus Lipiodol-Doxorubicin versus Doxorubicin-Loaded Beads for the Treatment of Hepatocellular Carcinoma. Korean J Radiol.

[38] Liu YM, Qin H, Wang CB, Fang XH, Ma QY [Comparision of different interventional therapies for primary liver cancer]. Zhonghua Zhong Liu Za Zhi.

[39] Chern MC, Chuang VP, Liang CT, Lin ZH, Kuo TM Transcatheter arterial chemoembolization for advanced hepatocellular carcinoma with portal vein invasion: safety, efficacy, and prognostic factors. J Vasc Interv Radiol.

[40] Tsochatzis EA, Fatourou E, O’Beirne J, Meyer T, Burroughs AK Transarterial chemoembolization and bland embolization for hepatocellular carcinoma. World J Gastroenterol.

[41] Brown KT, Do RK, Gonen M, Covey AM, Getrajdman GI, Sofocleous CT, Jarnagin WR, D’Angelica MI, Allen PJ, Erinjeri JP, Brody LA, O’Neill GP, Johnson KN Randomized trial of hepatic artery embolization for hepatocellular carcinoma using doxorubicin eluting microspheres compared with embolization with microspheres alone. J Clin Oncol.

[42] Hsieh CH, Liu CY, Shueng PW, Chong NS, Chen CJ, Chen MJ, Lin CC, Wang TE, Lin SC, Tai HC, Tien HJ, Chen KH, Wang LY Comparison of coplanar and noncoplanar intensity-modulated radiation therapy and helical tomotherapy for hepatocellular carcinoma. Radiat Oncol.

[43] Tang QH, Li AJ, Yang GM, Lai EC, Zhou WP, Jiang ZH, Lau WY, Wu MC Surgical resection versus conformal radiotherapy combined with TACE for resectable hepatocellular carcinoma with portal vein tumor thrombus: a comparative study. World J Surg.

[44] Kang J, Nie Q, R DU, Zhang L, Zhang J, Li Q, Li J, Qi W Stereotactic body radiotherapy combined with transarterial chemoembolization for hepatocellular carcinoma with portal vein tumor thrombosis. Mol Clin Oncol.

[45] Yu JI, Park HC Radiotherapy as valid modality for hepatocellular carcinoma with portal vein tumor thrombosis. World J Gastroenterol.

[46] Huang BS, Tsang NM, Lin SM, Lin DY, Lien JM, Lin CC, Chen WT, Chen WY, Hong JH High-dose hypofractionated X-ray radiotherapy for hepatocellular carcinoma: Tumor responses and toxicities. Oncol Lett.

[47] Xi M, Zhang L, Zhao L, Li QQ, Guo SP, Feng ZZ, Deng XW, Huang XY, Liu MZ Effectiveness of stereotactic body radiotherapy for hepatocellular carcinoma with portal vein and/or inferior vena cava tumor thrombosis. PLoS One.

[48] Benson R, Madan R, Kilambi R, Chander S (2016). Radiation induced liver disease: A clinical update. J Egypt Natl Canc Inst.

[49] Liang SX, Zhu XD, Xu ZY, Zhu J, Zhao JD, Lu HJ, Yang YL, Chen L, Wang AY, Fu XL, Jiang GL Radiation-induced liver disease in three dimensional conformal radiation therapy for primary liver carcinoma: the risk factors and hepatic radiation tolerance. Int J Radiat Oncol Biol Phys.

[50] Li XL, Guo WX, Hong XD, Yang L, Wang K, Shi J, Li N, Wu MC, Cheng SQ (2016). Efficacy of the treatment of transarterial chemoembolization combined with radiotherapy for hepatocellular carcinoma with portal vein tumor thrombus: A propensity score analysis. Hepatol Res.

[51] Yang M, Fang Z, Yan Z, Luo J, Liu L, Zhang W, Wu L, Ma J, Yang Q, Liu Q Transarterial chemoembolisation (TACE) combined with endovascular implantation of an iodine-125 seedstrand for the treatment of hepatocellular carcinoma with portal vein tumour thrombosis versus TACE alone: a two-arm, randomised clinical trial. J Cancer Res Clin Oncol.

[52] Li WW, Dai ZY, Wan HG, Yao LZ, Zhu J, Li CL, Wang XJ, Pan J, Chen LZ [Endovascular implantation of iodine-125 seeds strand and portal vein stenting followed by transcatheter arterial chemoembolization combined therapy with sorafenib for hepatocellular carcinoma with main portal vein tumor thrombus]. Zhonghua Yi Xue Za Zhi.

[53] Lau WY, Sangro B, Chen PJ, Cheng SQ, Chow P, Lee RC, Leung T, Han KH, Poon RT Treatment for hepatocellular carcinoma with portal vein tumor thrombosis: the emerging role for radioembolization using yttrium-90. Oncology.

[54] Moreno-Luna LE, Yang JD, Sanchez W, Paz-Fumagalli R, Harnois DM, Mettler TA, Gansen DN, de Groen PC, Lazaridis KN, KV Narayanan Menon, Larusso NF, Alberts SR, Gores GJ Efficacy and safety of transarterial radioembolization versus chemoembolization in patients with hepatocellular carcinoma. Cardiovasc Intervent Radiol.

[55] Yin J, Li N, Han Y, Xue J, Deng Y, Shi J, Guo W, Zhang H, Wang H, Cheng S, Cao G Effect of antiviral treatment with nucleotide/nucleoside analogs on postoperative prognosis of hepatitis B virus-related hepatocellular carcinoma: a two-stage longitudinal clinical study. J Clin Oncol.

[56] Tsuda Y, Kobayashi S, Tomimaru Y, Akita H, Hama N, Wada H, Kawamoto K, Eguchi H, Umeshita K, Doki Y, Mori M, Nagano H [Long-term survival of a patient with hepatocellular carcinoma with portal vein tumor thrombus treated with interferon- and 5-fluorouracil combination therapy]. Gan To Kagaku Ryoho.

[57] Huang G, Lai EC, Lau WY, Zhou WP, Shen F, Pan ZY, Fu SY, Wu MC Posthepatectomy HBV reactivation in hepatitis B-related hepatocellular carcinoma influences postoperative survival in patients with preoperative low HBV-DNA levels. Ann Surg.

[58] Bruix J, Raoul JL, Sherman M, Mazzaferro V, Bolondi L, Craxi A, Galle PR, Santoro A, Beaugrand M, Sangiovanni A, Porta C, Gerken G, Marrero JA (2012). Efficacy and safety of sorafenib in patients with advanced hepatocellular carcinoma: subanalyses of a phase III trial. J Hepatol.

[59] Bruix J, Takayama T, Mazzaferro V, Chau GY, Yang J, Kudo M, Cai J, Poon RT, Han KH, Tak WY, Lee HC, Song T, Roayaie S Adjuvant sorafenib for hepatocellular carcinoma after resection or ablation (STORM): a phase 3, randomised, double-blind, placebo-controlled trial. Lancet Oncol.

[60] Zhu K, Chen J, Lai L, Meng X, Zhou B, Huang W, Cai M, Shan H Hepatocellular carcinoma with portal vein tumor thrombus: treatment with transarterial chemoembolization combined with sorafenib a retrospective controlled study. Radiology.

[61] Lencioni R, Llovet JM, Han G, Tak WY, Yang J, Guglielmi A, Paik SW, Reig M, Y Kim do, Chau GY, Luca A, LR del Arbol, Leberre MA Sorafenib or placebo plus TACE with doxorubicin-eluting beads for intermediate stage HCC: The SPACE trial. J Hepatol.

[62] Qin S, Bai Y, Lim HY, Thongprasert S, Chao Y, Fan J, Yang TS, Bhudhisawasdi V, Kang WK, Zhou Y, Lee JH, Sun Y Randomized, multicenter, open-label study of oxaliplatin plus fluorouracil/leucovorin versus doxorubicin aspalliative chemotherapy in patients with advanced hepatocellular carcinoma from Asia. J Clin Oncol.

[63] Zheng JS, Long J, Sun B, Lu NN, Fang D, Zhao LY, Du N Transcatheter arterial chemoembolization combined with radiofrequency ablation can improve survival ofpatients with hepatocellular carcinoma with portal vein tumour thrombosis: extending the indication forablation?. Clin Radiol.

[64] Lu ZH, Shen F, Yan ZL, Li J, Yang JH, Zong M, Shi LH, Wu MC Treatment of portal vein tumor thrombus of hepatocellular carcinoma with percutaneous laser ablation. J Cancer Res Clin Oncol.

[65] Vibert E, Azoulay D, Cunha AS, Adam R, Samuel D, Castaing D Portal stenting for hepatocellular carcinoma extending into the portal vein in cirrhotic patients. J Surg Oncol.

[66] de Franchis R, Baveno VI Faculty Expanding consensus in portal hypertension: Report of the Baveno VI Consensus Workshop: Stratifying risk and individualizing care for portal hypertension. J Hepatol.

